# The Lords' Committee

**Published:** 1891-04-25

**Authors:** 


### THE LORDS' COMMITTEE.

The first sitting of the Lords' Committee after the Easter recess was occupied with evidence relating to the West-end Hospital. The noble Lords tried hard to find out why this institution is not in favour with the hospital world in general. M jor-General Mercer, the Chairman of the Board of Management of the West-end Hospital for Paralysis and Epilepsy, Welbeck Street, stated that that hospital was founded about twenty years ago by Dr. Tibbits. Since he had been Chairman it had been under repair, and was now being entirely rebuilt, and would be opened in June or July. There had been consequently no in-patients during the period of his chairmanship. There had, however, been many thousands of out-patients treated. He spoke also of the internal affairs of the hospital, and said that there had been some differences; but there had been a thorough re-organization. Dr. Tibbits, Hon. Medical Superintendent of the hospital, said there had been several bazaars and festivities, such as the Ice Carnival and the Coming Race, in aid of the hospital.

The Coming Race.

The former event realized a net profit of £1,400. But unfortunately the Coming Race was a loss which fell upon his shoulders. It had been his most earnest endeavour to ascertain why the Hospital Sunday Fund Council refused a grant to the hospital, but he was not successful. There was an intense jealousy of, and opposition to, this hospital. There was no objection to the massage treatment in itself, but there was to the treatment by nurses, as taking money out of the pockets of the medical profession. Mr. Alexander Dowell, the Treasurer and Hon. Sec., stated that he had found it necessary to keep the hospital always before the public in order to get the necessary funds. In two years he had been able to double the subscriptions—from about £330 to £650 a-year. Dr. Armitage, also connected with the same hospital, gave evidence in favour of special hospitals in certain cases, and particularly for paralysed and epileptic persons, who, if they went to the crowded outpatient departments of general hospitals, would have a very injurious effect upon others.

Mr. Stretch Dowse, M.D.Aberd., F.R.C.P.Edin., M.R.C.S.Lond., in examination by the Chairman, stated that he was now a general practitioner. He was formerly attached to the Charing Cross Hospital as resident physician, and he had also been attached to several special hospitals, amongst them to the West End Hospital for Nervous Diseases, where he was for nearly seven years. Just before he joined that hospital Dr. Tibbitts, the physician, was got rid of, for what reason he did not know; but after a lapse of six months a general meeting of the governors was held, and Dr. Tibbitts was re-elected to the post of physician, and his re-election completely broke up the organisation of the hospital, and all the medical men attached to the hospital and the members of the committee resigned. There was nothing but difficulty and disturbance from the first period of his connection with the hospital up to the time when he resigned. From the very first the hospital had not been properly organised, and they could not get it started on a fair, practical, and what might he might almost call honourable basis. The difficulties were administrative ones, and not connected with the treatment of nervous complaints. There were 24 Poor Law infirmaries in London, containing 12,032 beds, the larger number of which were perfect hospitals in every sense of the word; the nurses were efficient, well-trained, and highly respectable women; the medical superintendents were as a rule gentlemen of culture occupying a high professional status; and the matrons were usually women of high capacity, though a woman was sometimes appointed to this post with no other recommendation than a pretty face and a good figure, and in whom a guardian of the poor sometimes took a particular interest. In the main, these institutions were well officered and managed, but their organisation might be greatly improved, and what was known as Gathorne Hardy's Act, than which no more merciful Act of Parliament was ever passed, should be thoroughly and consistently carried out. The infirmaries were periodically visited by Poor Law inspectors in their customary perfunctory manner, and they were managed by gentlemen selected from the various boards of guardians, who were called managers; and this, to his thinking, was an unwise procedure which led to abuses. He would like to see these infirmaries managed and controlled by the London County Council, and ladies should have a share in the management. All classes of diseases, with the exception of infectious diseases and diseases of the mind, were treated in Poor Law infirmaries, and those who were suffering from lung diseases, cancer, consumption, or paralysis, who were admitted into hospitals only for a short time, found in these institutions a home for life, where they were visited once or twice a day by the medical superintendent, and treated with every consideration. It was certainly reprehensible that such rich fields for the investigation of almost every known form of disease should be left barren of teaching results to the medical and scientific world, whereas on the Continent similar institutions, such as the Salpêtrière in Paris, afforded valuable evidence of their scientific work. He wished it to be understood that his evidence in reference to this hospital was in no way personal to Dr. Tibbitts, who was very deaf and often conjectured things which made him very irritable. Nearly all special hospitals had been originated by medical men. In some cases, in all probability, it had occurred to a medical man who had failed in getting a hospital appointment, that he could make his mark by establishing a hospital for the treatment of some special form of disease, and many men of high position in London, such as Sir Spencer Wells, who had given special attention to the diseases of women, and he might say even Sir Morell Mackenzie, who had devoted himself to diseases of the throat, owed their success originally to their being attached to special hospitals.

Mr. Walter E. Scott, Secretary of St. Peter's Hospital for Stone, having described to the Committee the constitution, management, and system of account-keeping adopted at that institution, said that the average amount paid by each of their in-patients was about 3s. a-year, but if a person could not afford to pay anything he was not refused treatment.

Mr. Hurry Fenwick, F.R.C.S.Eng., Surgeon to St. Peter's Hospital, and also a member of the staff of the London Hospital, then deposed to the manner in which he had endeavoured to bring about co-operation between the general hospital and the special hospital. He had experienced no opposition, he said, from his colleagues at the London Hospital, but the system of co-operation was capable of further development.

